# Fake news threatens a climate literate world

**DOI:** 10.1038/ncomms15460

**Published:** 2017-04-20

## Abstract

As the challenges and environmental consequences of climate change manifest, the need for a society of science-literate citizens is becoming increasingly apparent. Achieving this, however, is no easy task, particularly given the proliferation of fake news and the seeds of confusion it can sow

The year 2015 may be regarded as the most environmentally significant year in history: the Paris agreement on climate change and the 2030 agenda for sustainable development are two unprecedented international agreements that together provide a roadmap to achieve a low-carbon, climate-resilient world and to eradicate global poverty. However, given the implementation of these agreements is largely dependent upon environmental laws and climate policies enacted by the elected governments of the world, many argue that a global society of climate and environmentally literate citizens is critical to success—an argument seemingly strengthened by the severe amendments to scientific programmes proposed by the recently elected US administration.

The rapid expansion of digital media has led to the wider dissemination of fake news articles, with the fast-paced nature of the modern newsfeed culture also encouraging less critical evaluation of news sources.

One route to knowledge empowerment is the wider dissemination of scientific information. UNESCO believes that open access to scientific literature is fundamental for scientific discovery, innovation and socio-economic development, and have highlighted it as key for realizing the majority of the 2030 sustainable development goals (http://www.unesco.org/new/en/communication-and-information/access-to-knowledge/open-access-to-scientific-information/). These sentiments are very much shared by the editorial team here at *Nature Communications*—all our articles have been freely available since January 2016 and are published under the least restrictive creative commons licence, allowing maximum re-use. However, is the provision of knowledge enough, particularly given the proliferation of misinformation in the modern-day culture of fake news?

While the concept has gained new heights in the wake of the recent US election, fake news has plagued climate and environmental science for decades. Influential misinformation campaigns, selective media exposure, fabricated controversies, alternative facts and false media balance (Boykoff, M. T. & Boykoff, J. M. *Glob. Environ. Chang.* **14**, 125–136 (2004)) have, in the view of many, manipulated scientific knowledge, sown seeds of confusion among the populace and threatened to derail environmental progress.

The public's awareness of the scientific consensus on human-caused climate change is a prime example of the consequences of scientific misinformation. With 97% of scientific experts in agreement that modern-day climate change is the result of human activity, the consensus is clear (Cook, J. *et al*. *Environ. Res. Lett.* **8**, 024024 (2013)). Yet, a 2016 survey by the Yale Program on Climate Change Communication showed that more than half of American adults are unaware that a consensus exists, with 28% believing a great deal of uncertainty remains (http://climatecommunication.yale.edu/visualizations-data/ycom-us-2016/).

Half of US audiences and two-thirds in the UK admit to not noticing the originating news brand responsible for providing their social media content (https://reutersinstitute.politics.ox.ac.uk/sites/default/files/Digital-News-Report-2016.pdf). Society's preference for like-mindedness and the echo-chamber effect generated by social media platforms can further perpetuate the problem. This is particularly concerning given that, by the age of 18, 88% of young adults claim to receive their news through Facebook and other social media (http://www.mediainsight.org/PDFs/Millennials/Millennials%20Report%20FINAL.pdf).

Wider visibility of scientific information alone is unlikely to resolve this issue. Research has shown that individuals' stable values, worldviews and political orientation rather than scientific knowledge are much stronger drivers of opinion on environmental risks such as climate change (Stern, P. C. *Nat. Clim. Chang.* **6**, 341–342 (2016)). Furnishing society with the skills necessary to distinguish fake news at a young age, before political allegiance is fixed and social networks are established, would therefore seem prudent. This is especially important in light of recent results indicating that 82% of US middle- and high-school students are unable to distinguish between a sponsored post and an actual news article on the same website (https://sheg.stanford.edu/upload/V3LessonPlans/Executive%20Summary%2011.21.16.pdf) and that a significant number of US teachers are failing to cite human activity as the underlying cause of climate change as part of their middle- and high-school curricula (Plutzer, E. *et al*. *Science* **351**, 664–665 (2016)).

The scale of the issue is daunting and a collective effort from all relevant stakeholders will likely be necessary to drive any discernible change, but positive signs are emerging. Educators are planning to install media literacy curricula to equip students with skills in critical thinking, independent verification and fact checking. Some media sectors are moving away from false balance and adopting a more interpretive approach where opinions are contextualized (Bruggemann, M. & Engesser, S. *Glob. Environ. Chang.* **42**, 58–67 (2017)), while Facebook and other social media platforms are collaborating with third-party fact-checking organizations in an effort to flag disputed content.

Increasing numbers of scientists are striving to engage with society regarding their research, but are in danger of falling into their own echo-chamber trap. Most scientists cite the sharing of information with colleagues as their primary objective, and those who do reach beyond their academic circle prioritize education and defence of scientific fact over building trust and establishing resonance with the public (Dudo, A. & Besley, J. C. *PLoS ONE* **11**, e0148867 (2016)). As publishers, we can offer some assistance in building this trust relationship through initiatives such as open and transparent peer review, which illuminate the intense scrutiny scientific works are subjected to prior to publication and also make the outcomes of publically funded research available to the taxpayer.

In order to fulfil their potential as effective educators, scientists need to be supplied with the skills, time and guidance necessary to take advantage of the technological tools available, and target engagement that establishes two-way dialogue and addresses society's perception of environmental risks rather than simply communicating more physical facts. The advent of a new interdisciplinary field known as translational ecology, seeking to arm scientists with the skills to translate environmental research into policy, represents a promising step in this direction (Schlesinger, W. H. *Science* **329**, 609 (2010)).

Successfully inoculating society against fake news is arguably essential if the hard-won environmental initiatives 2 years ago are to succeed. Should stakeholders collectively accept the responsibility to tackle this issue; perhaps 2015 will yet be celebrated as a year to remember.

Published online: 20 April 2016

